# Walk for life - the National Clubfoot Project of Bangladesh: the four-year outcomes of 150 congenital clubfoot cases following Ponseti method

**DOI:** 10.1186/s13047-016-0175-0

**Published:** 2016-11-09

**Authors:** Angela Margaret Evans, Mohommad Mamun Hossen Chowdhury, Mohommad Humayun Kabir, Md Fashiur Rahman

**Affiliations:** 1 Department of Podiatry, Lower Extremity and Gait Studies (LEGS) Research Program, La Trobe University, Bundoora, Melbourne, 3086 Australia; 2 Walk For Life, Road No 15, House 4, Ground Floor, Block D, Banani, Dhaka, 1213 Bangladesh; 3 Mymensingh Medical College Hospital, Mymensingh, Bangladesh

**Keywords:** Clubfoot, Ponseti, Relapse, Banglaesh, Outcomes

## Abstract

**Background:**

Congenital clubfoot deformity can cause significant disability, and if left untreated, may further impoverish those in developing countries, like Bangladesh. The Ponseti method has been strategically introduced in Bangladesh by a non-government organisation, Walk For Life (WFL). WFL has provided free treatment for over 17,500 Bangladeshi children with clubfeet since 2009, sustained by local ownership, and international support. This study assesses the 4-year results in children for whom treatment began before the age of 3 years.

**Methods:**

A centrally located WFL clinic at Mymensingh Medical College Hospital (MMCH), representative of the larger WFL clinics, which treats >100 cases annually, was reviewed. In 2015, 99 of the 147 eligible subjects who had begun treatment in 2011 were available for follow up. Specific assessment tools enabled evaluation of parent satisfaction, gait function, and relapse cases.

**Results:**

Results for 99/147 cases were returned after four years: 72 males, 27 females. Typical clubfeet comprised 98/99 of cases, and 55/99 were bilateral. The tenotomy rate was 80 %. Brace use after 3 months was 90 %, at 12 months was 65 %, and at 4 years post treatment was 40 %. Functionally, 98/99 of children could walk and run (99 %). Relapsing deformity was found in 13 %. Relapse severity varied: eight were flexible and partial, five were rigid. Half of the children lost to follow were due to changed phone numbers. While parents were very happy with their child’s feet (97 %), a materials cost of 3000 Taka ($US40) was deemed unaffordable by 60 %.

**Conclusions:**

The 4-year outcomes after Ponseti treatment for clubfoot deformity, showed that 99 % of children available for follow up, were walking independently. The relapse rate was low. Parent satisfaction was high, but those whose children required further treatment were less satisfied.

## Background

It is estimated that 5000 Bangladeshi children are born with a clubfoot deformity each year, with incidence approximated at 1: 900 births (www.walkforlifeclubfoot.org) [1]. Neglected clubfoot deformity inevitably leads to long-term disability for the child, limited employment opportunities, limited socialisation, and extenuated family poverty [2]. As reported previously, treatment is generally unaffordable for the families, and only due to the beneficence of ‘Walk for Life’ – the sustainable and National Clubfoot Project in Bangladesh since 2009 (WFL) - and its donor and volunteer base, has the global ‘gold standard’ Ponseti method for clubfoot correction been implemented and averted long-term disability in over 17,500 children [3].

Paediatric clubfoot, *talipes equino varus*, is one of the most significant congenital orthopaedic deformities [4], with no single known cause. Genetic studies have now identified involvement of specific and varying chromosomes and transcription factors [5].

Management with splints, binding, and plaster casts has been evident across hundreds of years, and in the 20th century these conservative measures were largely subsumed by surgical correction, and notably the posterior medial release (PMR) [6, 7]. The PMR is a joint invasive procedure, which severs to lengthen the tight soft tissue structures on the medial and posterior aspects of the infant clubfoot [8].

In the 21st century, surgical correction of clubfeet has been firmly denounced [9]. Retrospective reviews and prospective studies have both shown the poor outcomes, in terms of pain and function, resulting from the PMR surgical procedure [10].

The non-surgical Ponseti method has been extensively investigated and found to give the best clinical outcomes, and also to be a more cost-effective, when compared to surgery [4, 11]. The Ponseti method is now regarded as the global ‘gold standard’ for treating congenital clubfoot deformity, and has been disseminated across the world, and especially the developing world where most affected children live, and hence need is greatest [12].

WFL has grown quickly and currently supplies administration, professional staff, materials and equipment in 39 clinics across the country, so that no child has to travel more than 60 km to access corrective treatment. With the support of the Minister for Health, WFL has expanded so that children can be treated at the earliest possible age.

Previously we reported the mid-term results of this large-scale project indicating its extraordinary effectiveness with 99 % of treated children walking independently two years after treatment [3]. However, given the tendency for clubfoot to relapse following initial correction, the need for both continued and longer-term outcome assessments was also identified, directing this further evaluation, with particular emphasis on any relapsing deformities.

The aim of this study was to review the results for Bangladeshi children with congenital clubfoot deformity 4 years after being treated with the Ponseti method, with treatment commencing before the age of 3 years. A further and key objective was to explore any relapsing deformities more specifically, which may better inform not only WFL in Bangladesh, but also other clubfoot clinics around the world.

## Methods

### Study population

A total of 151 children with congenital clubfeet attended the MMCH clinic in 2011, with treatment commencing before the children were aged 3 years.

### Inclusion and exclusion criteria

- Inclusion criteria
- Patients with clubfeet who began treatment at the MMCH clinic in 2011, before the age of 3 years.
- Exclusion criteria

Patients with clubfoot associated with a syndrome, those who had previous surgery for clubfoot, or those referred to other hospitals.

### Sampling technique

To facilitate the logistics of the study (travel distances between clinics, workload of the examiner, financial costs), one WFL clinic was selected. The WFL clinic at Mymensingh Medical College Hospital (MMCH), situated 120 km north of Dhaka, with more than 100 clubfoot cases seen annually, was selected to represent the WFL clinic network, being a large catchment clinic, conveniently located and permanently staffed. In 2011, WFL had 31 clinics across Bangladesh, which treated 2004 clubfoot cases (with 3077 clubfeet). Seven of the 31 WFL clinics treated >100 cases in 2011 Range: 100 – 160 cases), with a further 10 clinics treating 50–100 cases, and 14 newer clinics treating <50 cases/2011.

At MMCH, 151 clubfoot cases were enrolled in 2011, and at review in 2015, 103 patients who had completed treatment, were located for follow up. Excluding four syndromic cases, there were 99 cases meeting the study criteria. These families were recalled or visited for this study, and reimbursed if transport costs were incurred.

### Research instruments/data collection tools

The previously developed Bangla clubfoot assessment tool was used to evaluate treatment outcomes, as was previously performed when addressing 2-year outcomes [3, 13]. The specific evaluation of any relapsed cases, used an additional assessment tool, after Bhaskar [14] (Table 1).

**Table 1:** Relapse assessment protocol (after Bhaskar) [14]

LEFT	RIGHT	Description
1A/2A	1A/2A	Reduced ankle dorsiflexion: <15 to 0 (1A) Fixed ankle equinus: <0 (2A)
1B/2B	1B/2B	Gait supinated, dynamic forefoot adduction/supination – flexible relapse (1B) Fixed adduction of forefoot - curved and rigid relapse (2B)
3	3	Two or more fixed deformities*

Relapse assessment was performed in two steps

1. Examine ankle range, with knee extended

2. Watch gait for supination

*Fixed ankle equinus and adduction and cavus (partial relapse)

*Heel varus and ankle equinus, midfoot cavus, forefoot adduction (total relapse)

The Bangla clubfoot assessment tool enables efficient and relevant evaluation of outcomes. It comprises three domains to assess parent satisfaction, gait and function, clinical examination, and has been found to be repeatable [3]. Appending the relapse evaluation component of the Bhasker tool, enabled structured assessment of the forefoot and heel positions, and identification of adduction or varus respectively, indicative of deformity relapse [14].

Demographic data, family history, and housing construction (as a proxy assessment of affluence), were included as a part of the patient’s wider clubfoot history.

### Data collection

One of the authors (MC) collected the data using face-to-face interviews with the parents, and examination of the patients.

The Bangla clubfoot assessment tool addresses three domains: parent satisfaction, gait and function, and clinical assessment. The subjective elements of parents’ assessment addressed satisfaction with their child’s treatment (five questions – Table 2). The gait and clinical aspects of assessment included the children’s physical ability to squat, walk, run, and use stairs, (four items – Table 2) and objective assessment of heel position and ankle range (two items – Table 2).

**Table 2:** The Bangla clubfoot tool combined three domains of parent satisfaction, gait, and clinical examination (total score from 11 points, then rated as a percentage)

A. Parental rating	Yes +1	Don’t know 0	No −1	Mean scores (%)	‘Rating’ #
1. Happy with child’s feet?	96	3	0	97	
2. Recommend to others?	94	5	0	95	
3. Does child play with others?	97	2	0	98	
4. Does child wear shoes of choice?	55	33	11	44	~
5. Does child have pain?	91	4	4	85	
Parental Rating sub score				4.19/5 (84 %)	‘Good’
B. Gait assessment	Yes +1	Not fully/with assistance 0	No −1	Mean scores (%)	
6. Squatting	84	14	1	84	
7. Walking	98	1	0	99	
8. Running	98	1	0	99	
9. Up/down steps	95	4	0	96	
Gait assessment sub score				3.75/4 (94 %)	‘Very good’
C. Clinical examination	Valgus +1	Straight 0	Varus −1	Mean scores (%)	
10. Heel position - left	3	60	13	13	
Heel position - right	4	65	11	9	
	>0 dorsiflexion +1	0/90 degrees 0	<0 dorsiflexion −1	Mean scores (%)	
11. Ankle range - left	65	11	0	79	
Ankle range - right	70	8	2	85	
Clinical examination sub score				1.30/2 (65 %) ^a^	‘Fair’
Total score				9.24/11 (84 %)	‘Good’

**#** Ratings

Very good: 85–100 %, Good: 70 – 85 %, Fair: 60–70 %, Poor: <50 %

**~** many children were did not have shoes

^**a**^Note: Scores for bilateral cases were halved to achieve same scale/foot for section C/clinical examination

Following assessment with the Bangla clubfoot tool, cases identified as having a relapsing clubfoot deformity from the objective assessment of heel position and ankle range (Table 2), were further assessed using the Bhaskar relapse tool. This tool evaluates heel position, forefoot position in stance and gait [14]. The flexibility or rigidity of each component is also noted (Table 3).

**Table 3:** Relapse assessment results

Relapse L/R/Both	Ankle L	Ankle R	Forefoot L	Forefoot R	Combined L	Combined R	Relapse type
1		2		2		3	Total, R
2		1		2			Partial, R
3		1		2			Partial, R
4	1	1	2	2			Partial, B
5		2		2		3	Total, R
6			1	1			Partial, L
7	2	2	2	2	3	3	Total, B
8		1		1			Partial, R
9	1	1	1	1			Partial, B
10	1		1				Partial, L
11	1	1	2	2			Partial, B
12	2	2	2	2	3	3	Total, B
13	2	2	2	2	3	3	Total, B

There were 13/99 children showing relapse signs four years after their treatment. Of these, only 5 cases were more fixed, and 8 cases were flexible and functional feet

•Fixed ankle equinus and adduction and cavus (partial relapse)

•Heel varus and ankle equinus, midfoot cavus, forefoot adduction (total relapse)

Several proxy indicators of affluence were assessed. Parents were asked if a materials cost would have been affordable for their child’s treatment. Basic demographic factors included: the father’s monthly wage and occupation, the number of earning members/household, housing type.

### Data management and analysis

All examination findings were entered into a database for statistical analyses using constructed data sets in a Microsoft Excel 2000 (Microsoft Inc., Redmond, Washington) software package. Further analysis utilized IBM SPSS Statistics (version 22) software. Descriptive statistics and frequency data were obtained to explore and summarise the demographic, case records, and assessment findings. Non-parametric statistical testing (Spearman’s rho) was used to analyse univariate correlations between categorical data. Significance levels were designated as *p* < 0.05 or *p* < 0.001. Significant univariate correlations were subjected to logistic regression modelling, primarily to explore predicted probability for clubfoot relapse.

## Results

The 48/147 (32.6 %) eligible children who commenced treatment in 2011, but were lost to follow up in 2015, The 48 children lost to follow up were all accounted for as follows: 27 were unable to be contacted due to discontinued phone numbers; 15 had withdrawn from treatment during the casting phase; one had withdrawn during the brace phase; one was lost to follow up when parents separated; two had failed to attend any review appointments; two had died of pneumonia (both children were in the brace phase).

**Fig. 1:**
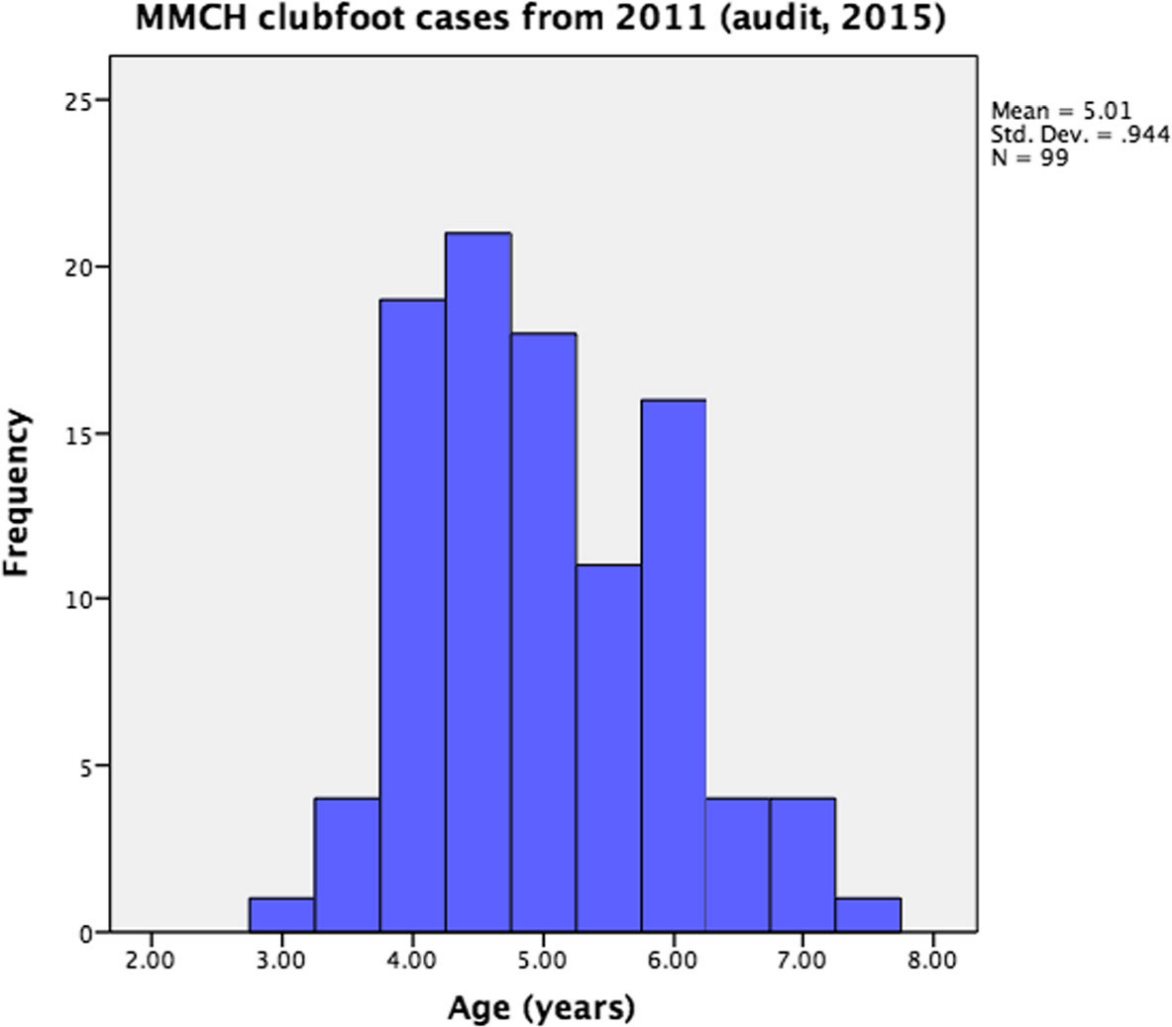
The average age of the children returning for review was five years

Of the 151 children who commenced treatment for congenital clubfoot deformity at MMCH in 2011, four were ineligible for the current study due to having syndromic clubfoot. Subsequently, 147 children were eligible for inclusion, of which 99 were available for follow up (Table 4, Fig. 1).

**Table 4:** Descriptive data for the 99 cases available for follow up review four years after Ponseti method clubfoot treatment

Variable	Mean	Std Deviation	Minimum	Maximum	Range
Age (years)	5.01	0.94	3.00	7.50	4.50
Age at first cast (years)	1.01	0.78	0.20	2.40	2.20
Initial Pirani L	4.64	1.01	1	6	5
Initial Pirani R	4.71	0.93	1	6	5
No. casts pre tenotomy	4.89 (median = 5 casts)	2.39 (median = 2 casts)	1	17	16

For the available study sample of 99 children, gender distribution was male 72: female 27. Clubfoot type was: typical - 98, complex - 1. The foot affected was: left only - 17, right only - 27, both feet - 55.

A family history of clubfoot deformity was reported in 9/99 (9.1 %).

Further treatment had been provided in 14 children, which consisted of re-casting/brace in 12 children, and referral (surgical) in two children.

The mean age of children at the application of the first corrective cast was 1.01 years (range 0.20 – 2.40 years). Age was also categorised as follows: 0 to 3 months – 31/99, 4 to 12 months – 26/99, 1 to 2 years – 28/99, 2 to 3 years – 14/99. Overall 57/99 children commenced treatment before 12 months of age and the onset of walking.

The number of corrective casts required before the percutaneous achilles tenotomy (PAT) was performed were reviewed: 11/99 had 1–2 casts pre PAT (11.1 %), 74/99 had between 3 and 6 casts (74.7 %), 17/99 had 7 – 11 casts (17.2 %), and 1 child had 17 casts (1.0 %).

PAT was performed in 80/99 (80.8 %), not performed in 19/99 (19.2 %). One child had multiple tenotomies.

Brace use for the first 3 months was reported to have been full-time in 90/99 (90.9 %) and less than the specified full-time (23 h/day) in 8/99 (8.1 %) children. At 12 months brace use for the required 12 h was reported in 64/99 (64.6 %), and less than 12 h in 34/99 (34.3 %) children. At the time of assessment, 4 years after treatment began, 40/99 (40.4 %) reported that they were still using the brace, and 59/99 (59.6 %) reported having no brace use.

Results from the Bangla clubfoot tool assessment, showed that parental satisfaction levels were good (84 %), as seen in Table 2, and potentially confounded by shoe use being inapplicable for some younger children. Functionally, 98/99 (98.9 %) of the children were able to walk, 98/99 (98.9 %) children could also run, squat fully 84/99 (84.8 %), and manage steps independently 95/99 (95.9 %). Some children required assistance for negotiating steps 4/99 (4.1 %), unable to fully squat 14/99 (14.1 %). Two children could not manage to squat.

Clinical assessment of the heel found a varus position in 13/99 (left, 13.1 %) and 11/99 (right, 11.1 %), which is suggestive of relapsing deformity. Likewise, 11/99 (left, 11.1 %) and 8/99 (right, 8.1 %) ankles showed range of motion that reached planti-grade only. In two children, (2.0 %) ankles were found to have insufficient dorsi-flexion range of motion to reach planti-grade.

When asked if the materials cost of 3000 Taka ($US 38.48) would have been affordable for their child to receive this treatment, 26 parents replied yes (41 %), 72 parents replied no (59 %). One parent was unclear in their response.

As further and proxy indicators of affluence, several demographic factors were included: the father’s monthly wage was categorised according to occupation, the number of earning members/household, housing structure material (Tables 5, 6 and 7).

**Table 5:** Father’s monthly wages and occupation categories

1	Guard	Tk 5000 (US$ 60)
	Day labourer	
	builder	
	Shop worker	
	Fisherman	
	Hawker	
	Rickshaw puller	
2	Garment or factory worker	Tk 10,000 (US$ 120)
	Helper to bus driver	
	Mechanic	
	Hospital porter	
	Farmer	
	Imam	
	Laundry worker	
3	Car driver	Tk 20,000 (US$ 240)
	owns own business	
	Clerk in office	
	Teacher	
	Police officer	
	Working for NGO	
4	Working abroad	Tk 20,0000 (>US$ 240)
	Banker	
	Doctor	
	Army	
	Engineer	

From the 99 returning cases, there were 16 in category 1, 38 in category 2, 43 in category 3, and 2 in category 4

**Table 6:** The majority of families had one earning member, usually the father

No. earning members per family	*n* = 99
1	69
2	25
3	3
4	1
5	1

If the earning family member becomes ill or injured, another (usually the mother) will have to work more, be this paid/unpaid. Visits to clinics become much less likely under such circumstances

**Table 7:** Housing material was collected as a proxy measure of family affluence

Housing materials	*n* = 99	Affluence level
Brick	13	1 (highest)
Tin	78	2
Bamboo/wood	2	3
Mud	6	4 (lowest)

Relapse cases (*n* = 13) were assessed specifically as shown in Table 3, using the criteria to address forefoot adduction, ankle range and flexibility of these positions, after Bhaskar et al. [14]. Two variables returned significant correlation with relapse: Further treatment (rho = 0.862**), Brace use at 12 months (rho = 0.284**), but were not significant after any further regression analyses. The small sample size of relapsed cases is likely to be relevant to these statistical results.

Table 8 displays the variables which returned significant correlations and the strength of the relationships.

**Table 8:** Correlations were repeatedly found between the need for further treatment and various parent ratings, as well as children’s mobility

Moderate strength correlations	0.50 – 0.70, *p* ≤ 0.05* or 0.01**
Age at first cast *and* the need for further treatment	rho = 0.597*
Number of casts before the tenotomy *and* the need for further treatment	rho = 0.550*
Number of casts before the tenotomy *and* the ability to walk/run	rho = −0.513**
Brace use at 3 months *and* the ability to manage stairs	rho = −0.596**
Walking *and* the need for further treatment	rho = −0.679**
Running *and* the need for further treatment	rho = −0.679**
Further treatment *and* parent happiness	rho = −0.679**
Strong correlations	>0.70, *p* ≤ 0.05* or 0.01**
Parent happiness *and* the ability to manage stairs	rho = 0.862**
Parent happiness *and* play	rho = 0.700**
Parent satisfaction domain *and* normal shoe use	rho = 0.907**
Ability to squat *and* need for further treatment	rho = −0.703**
Ability to walk or run *and* play	rho = 0.703**
Need for further treatment *and* pain	rho = 0.706**
Perfect correlations	=0.100, *p* ≤ 0.01**
Parents’ rating of child’s play *and* the need for further treatment	rho = −0.100**
Ability to manage stairs *and* need for further treatment	rho = −0.100**

*p < 0.05 and **p < 0.001

Data was grouped, and correlation strength and direction was interpreted. Findings revealed that parent satisfaction was strongly associated with: children having straight feet (forefoot adduction correlated inversely with satisfaction, rho = 0.703**); and children being able to wear normal shoes (rho = 0.907**). Parents were less happy when children were unable to manage stairs (rho = −0.862**), and happy when children could play well (rho = 0.703).

Similarly, the need for further treatment (14/99 cases) following the Ponseti method was a strong, significant and inverse correlate with: the Bangla tool gait domain (rho = −0.762**); right ankle range (rho = −0.804**); ability to squat (rho = −0.703**).

Atypical *or complex* clubfeet [15] were reported in 5/147 (3.4 %) children commencing treatment in 2011: 1/99 returning children and in 4/48 children not examined 4 years after treatment. This is proportionally less than previously found [3].

## Discussion

This study focused on the four-year physical and functional outcomes of children who were treated by WFL for congenital clubfoot, prior to 3 years of age. The Bangla clubfoot tool had been previously developed for the unique conditions and ongoing evaluation of clinical outcomes. The relapse tool was incorporated for the first time [14].

The sample met demographic expectations, as most children were boys with bilateral clubfeet. Less than 10 % reported a family history of clubfeet, comparable to our previous findings. The high rate of tenotomies being performed was notable at 80 %, and was similar to our previous findings of 76 % at 2-year follow up [1, 3]. The initial brace use at 3 months post treatment was commendable at 91 % for the stipulated 23 h per day. However, we again found the use of the boots and brace tapering with time, with only 65 % using for the required 12 h/day after 12 months, in this 4-year follow up. This is considerably less than the previously reported 80 % use at 12 months, as found at the 2-year follow up [1, 3]. At the time of review less than half of the returning cases were using the brace (40 %), which is a well-reported finding in other studies [16–18].

**Fig. 2:**
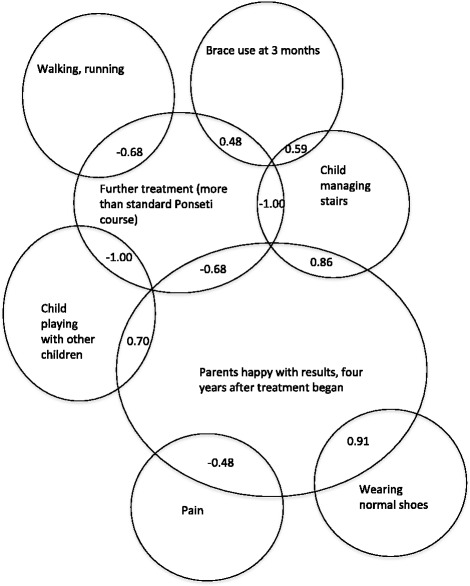
Schematic diagram showing related treatment factors and significant bivariate correlations with parent satisfaction (*p* < 0.01; *rho*)

There were many significant univariate correlations with parents’ ratings of outcomes (Fig. 2). This study has identified that the need for further treatment following the initial Ponseti treatment course is a very significant indicator of both poor functional outcome for the child, and unhappy parents. The cases requiring further treatment, were associated with: reduced ankle range, inability to squat, poorer gait and function, and pain. We again found that children who begin treatment at a younger age are less likely to require further treatment [1, 3].

The low relapse rate is a positive outcome overall, but did prevent identification of any independent variables which may be predictive of relapsing deformities. This will continue to be investigated in a future, and larger study sample.

Given the scale of WFL, the consistent training of staff and consistent replication of all clinic set ups, it is not unreasonable to extrapolate the 13 % relapse rate across the >17,500 clubfoot cases enrolled in WFL clinics to date. Potentially, simple modelling indicates that 2275 (13 %) cases could be expected to show some form of relapsing deformity, and 15,225 (87 %) would remain fully corrected at a 4-year review. Furthering the extrapolation (see Table 3), of the 2275 notional relapse cases, only 875 would be fixed and more problematic, with 1400 being flexible relapses, which are usually functional (viz. adducted but still plantigrade). Given the 30 % relapse level reported elsewhere [19], and acknowledging the uncertainty of the extrapolation, there is still fair indication that WFL results appear very good.

Comparison with other studies is relevant, yet needs to be appreciated for the inherent differences in settings. It is reported that 113 of 193 United Nations countries have clubfoot projects [12]. Given the large number of clubfoot projects using the Ponseti method globally, the results of treatment after 4 years are scant, with initial and short -term results well-reported [20, 21]. Recent studies which do afford comparison include a 4-year review in Norway of 116 cases (162 clubfeet), which found that only five cases had required extensive surgery to that point (some re-casting, repeated tenotomies did occur) [22]. Better brace use has been repeatedly found to reduce relapse [18, 23–25] and has been reported in a 6-years follow up study of 189 cases (279 ft) [26]. This study found that surgery was more likely if brace use stopped before 28 months of use, versus cases wearing the brace for 33 months or more were less likely to have required surgery [26]. Brace use to ‘school age’ (approximating ages 5 to 6 years) is usually advised as part of the Ponseti maintenance phase.

Congenital clubfoot deformity is a challenging condition to manage, and relapses following initial correction using Ponseti method have been reported as 30 % [19] and a fact intrinsic to the condition per se [27]. That said, better brace use is well documented as being associated with lower relapse rates, which in turn is associated with parents continuing to ensure that the children wear the braces [28, 29].

What is very clear from the findings of this study, is the need to work with the parents if optimal results are to be obtained with the Ponseti method [30]. The parents need to be informed about the treatment course, and all that is involved, before/as the first cast is applied. Sequential pictures of the corrective process, and maintenance brace use can be very useful, and especially necessary in developing countries like Bangladesh, where literacy approximates 30 % [31]. Support and follow up during the brace period is regularly undertaken by WFL, but travel to clinics can be arduous, expensive, and time-consuming.

The management of children with clubfeet in the WFL clinics has, to date, been provided at no cost to the parents, which is challenging for the sustainability of this project. By developed world standards the cost of clubfoot treatment at WFL in Bangladesh is very small (total cost approximates AUD$120, variable with exchange rate fluctuation), yet over half of parents reported that they would not have been able to afford to pay even a basic materials cost (approx. US$40) for their child’s treatment.

As previously reported [1, 3, 17, 32], we found that children beginning treatment at a younger age, had better outcomes overall, and were less likely to require additional treatment following the standard Ponseti treatment course. Parents were understandably displeased with any further treatment requirements, and less functional outcomes for their children (as occurred in 14 cases reviewed after 4 years). The complex issue is that parents may inadvertently contribute to sub-optimal outcomes, and hence the need for further treatment. Our clinical experience (unpublished) shows that missed appointments, not attending to broken casts, and poor brace use are common factors contributing to incomplete clubfoot correction, and simultaneous difficulty and frustration at a lengthening treatment process.

There are usually reasons for parents not following the Ponseti correction course adequately, and given the poverty of people in the catchment area of MMCH, many times this is simply beyond the demands/resources of parents eg bus fares, caring for other children, work demands, illness. In other instances, it is possible that parents have not sufficiently understood the treatment course, especially the ongoing bracing needs, or are advised otherwise by someone of greater influence than the treating clinicians viz a grandparent or other local elder.

### Limitations of the study

These results relate to 4 years post treatment, in children still reliant upon continued use of the boots and brace to prevent relapse.

For convenience and logistics, one of the large WFL clinics was selected. Given the standardised training of the WFL physiotherapists, doctors and assistants and the consistent use of the Ponseti method, it is reasonable to infer that results will be largely representative of those across all WFL clinics, but clearly this is not certain.

Further, it is possible that the local examiner (MC), who was neither a blinded assessor, nor neutral as a WFL staff member, may have introduced assessment bias.

The outcomes in 99/147 young children reviewed after 4 years of being treated with the Ponseti method for clubfoot deformity showed very good results. There was high percentage of well-corrected feet, and 98/99 of the children were walking and running independently. However, these results must be tempered with the 48 cases lost to follow up, a common issue with case reviews.

The assessment tools enable ongoing monitoring of the children treated for clubfoot deformity by WFL [13, 14]. Without the patronage of WFL, almost 60 % of these children would not have received treatment, an issue that underpins the sustainability of this demonstrably beneficial clubfoot project in Bangladesh.

## Conclusions

WFL in Bangladesh is demonstrably successful and beneficial for the children affected by congenital clubfoot deformity. Follow up assessment was availed in two-thirds of cases, in children with mean age of 5 years, and found that 99 % were walking and running, 4 years after treatment.

This study also found a low relapse rate in children assessed 4 years post treatment utilising the low cost, non-surgical Ponseti method of correction, and that approximately half of the clinical relapses were functional.

WFL is a large-scale, well- structured clubfoot aid programme based on consistent training and updating of retained staff. The results across the multiple clinics in Bangladesh are likely to be consistent with those found at the clinic reported in this study.

Managing any paediatric condition requires good cooperation of the parents to gain best outcomes. It is very apparent from the findings of this study, that disruption of the treatment course yields lesser results, protracts the treatment process, and reduces parent satisfaction. In Bangladesh, where adult literacy is low, whilst daily living and transport difficulties are high, it is an ongoing challenge to optimally meet the needs of both children requiring treatment for clubfoot deformity and their parents.
